# Disparities in demand for COVID‐19 hospital care in the United States: Insights from a longitudinal hierarchical study

**DOI:** 10.1002/hsr2.441

**Published:** 2022-01-07

**Authors:** Wolfgang Messner

**Affiliations:** ^1^ Darla Moore School of Business University of South Carolina Columbia South Carolina USA

**Keywords:** COVID‐19, health insurance, hospitalization, pandemic, racial/ethnic disparities

## Abstract

**Background and aims:**

This study examined disparities in hospitalization for COVID‐19 within the U.S. by racial and ethnic groups, health insurance status, and social support structure.

**Methods:**

Using publicly available ecological case and contextual data from July 2020 to April 2021, a longitudinal hierarchical model for the 51 U.S. states was constructed.

**Results:**

Racial/ethnic disparities were observed, such as that hospitalization rates were higher in states with a higher percentage of Black (*β* = .002, *P* = .009) and American Indian or Alaska Native persons (*β* = .003, *P* = .03). Conversely, lack of health insurance was related to a lower hospitalization rate (*β* = −.005, *P* = .002), and so was a stronger social support system (*β* = −.015, *P* = .05).

**Conclusion:**

These differences suggest disparities in COVID‐19 incidence, symptom severity, and demand for hospital care. Understanding how they contribute to geographic differences in hospitalization can help guide public health decisions and resource allocation to address COVID‐19‐related health inequalities.

## 1 INTRODUCTION

First reports of pneumonia of unknown etiology emerged in Wuhan, China, on December 31, 2019. The extremely contagious virus was identified as SARS‐CoV‐2, causing the disease COVID‐19. While it spread quickly around the globe, differences in the outbreak existed between and within countries.^1^, ^2^, ^3^ The pandemic strained hospital resources, and necessitated the need for models forecasting patient care demands. Because a community's susceptibility to any virus is determined by a variety of factors, including biological determinants, demographic profiles, and contextual factors,^4^ in a country as diverse as the U.S., there was likely considerable intra‐country variation. While first progress in understanding disparities in hospitalization rates for COVID‐19 by racial and ethnic groups has been made,^5^, ^6^, ^7^, ^8^ differences in demand for care by health insurance status and social support structure are not well understood. Using ecological data from 51 U.S. states during July 2020 to April 2021, this study constructed a longitudinal hierarchical model to assess the COVID‐19 hospitalization rate in relation to confirmed cases by race/ethnicity, health insurance status, strength of the social support system, and other contextual factors.

## 2 MODELS AND METHODS

COVID‐19 case, mortality, and hospitalization data were obtained from USAFacts and the U.S. Department of Health and Human Services (HHS).^9^, ^10^ Since January 22, 2020, USAFacts collects daily positive cases from state public health websites. Presumptive positive cases are counted as confirmed cases, in line with reporting by the Centers of Disease Control and Prevention (CDC); cases are assigned to the state where the person was diagnosed. Deaths are counted as COVID‐19 related if the disease played a significant role in causing death. The data is taken directly from state and local agencies; if a state reports both the location of death and the location of residency, the death is attributed to the location of residency. HHS provides state‐aggregated data for COVID‐19 hospital admission and utilization derived from facility‐level reports; the data is consistent starting from July 15, 2020. Both confirmed and suspected, adult, and pediatric admissions are counted. In this study and for all timeseries variables, a one‐week trailing average was used during the period July 21, 2020 to April 10, 2021; a nine‐day time lag accounted for the average time between diagnosis and hospital admission.^11^ Patient race and ethnicity variables were categorized as Black, Hispanic, American Indian or Alaska Native (AIAN), and Asian. Uninsured includes those without health insurance and those who have coverage under the Indian Health Service only. Social support is a social capital index, which covers emotional support, trust in neighbors, doing favors for neighbors, and average number of friends. Disabled, diabetes, obese, and smoking are percentages of the population with these conditions. Rural captures the population percentage living in a non‐urban area; median age is the state's median age. Spending is the state/local government spending per capita. These state‐level variables were sourced from the Social Capital Project, published by the U.S. Congress Joint Economic Committee.^12^

The study used a two‐level linear model for longitudinal data, with restricted maximum likelihood. It tracks hospitalization across time (index *t* in the below equations) for the same set of states (index *i*), that is, multiple observations *t* are nested within a single entity *I*.^13^ When there is longitudinal data with temporal autocorrelation, a failure to accommodate a nested data structure would lead to improper statistical inferences.^14^, ^15^ At level 1, the model compared hospitalization with COVID‐19 cases and the pandemic threat level, which is operationalized with the number of deaths:

$${\text{Hospitalization}}_{\mathrm{ti}}=\pi_{0i}+\pi_{1i}*{\text{Cases}}_{\mathrm{ti}}+\pi_{2i}*{\text{Deaths}}_{\mathrm{ti}}+e_{\mathrm{ti}}.$$

Then, at level 2, the model controlled the association of cases and hospitalization for state‐level variables:

$$\begin{array}{l} \begin{array}{l} \pi_{0i}=\beta_{00}+r_{0i}\\ \pi_{1i}=\beta_{10}+\beta_{11}*\left({\text{Black}}_{i}\right)+\beta_{12}*\left({\text{Native}}_{i}\right)+\beta_{13}*\left({\text{AIAN}}_{i}\right) \end{array}\\ \;+\beta_{14}*\left({\text{Asian}}_{i}\right)+\beta_{15}*\left({\text{Rural}}_{i}\right)+\beta_{16}*\left({\text{Uninsured}}_{i}\right)\\ \;+\beta_{17}*\left(\text{Median}\;{\mathrm{Age}}_{i}\right)+\beta_{18}*\left({\text{Disabled}}_{i}\right)+\beta_{19}*\left({\text{Diabetes}}_{i}\right)\\ \;+\beta_{110}*\left({\text{Obese}}_{i}\right)+\beta_{111}*\left({\text{Smoking}}_{i}\right)+\beta_{112}*\left({\text{Spending}}_{i}\right)\\ \;+\beta_{113}*\left({\text{Social Support}}_{i}\right)+r_{1i}\\ \pi_{2i}=\beta_{20}+r_{2i} \end{array}$$

The level‐2 variables were not modeled to influence *π*_0i_, because cases and deaths are baseline changes; hospitalization does not fluctuate without changes in those level‐1 variables. Deaths as a covariate represents the danger associated with the pandemic, in concordance with terror management theory^16^ and the terror management health model.^17^ Grounded in evolutionary theory, terror management theory offers predictions for how people behave in response to fear associated with mortality. It acknowledges that humans are unique in their use of conscious thought processes, and proposes that humans exhibit a biological predisposition toward self‐preservation and reproduction.^18^ Humans change behavior in novel circumstances, with hospitalization being such a behavioral health response to a threat. All predictors were centered around the group mean at level 1, and grand mean at level 2. *P*‐values <.05 were considered statistically significant. SPSS (version 27; IBM) and HLM (version 7.3; Scientific Software International) were used to conduct all analysis.

### 2.1 Statistical results

**FIGURE 1:**
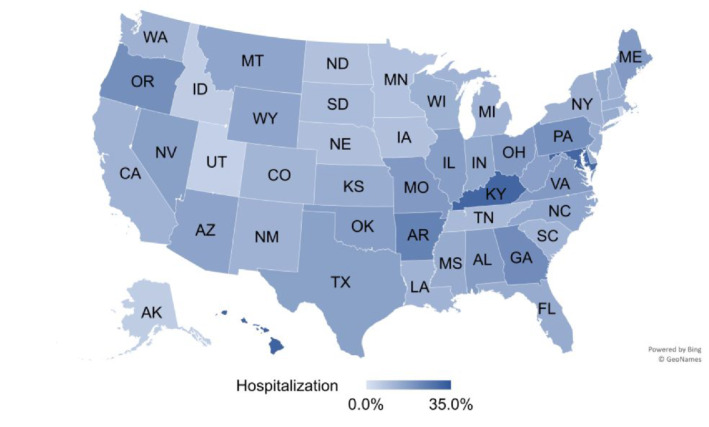
COVID‐19 hospitalization rates at state level. This map shows the state‐level averages of the COVID‐19 hospitalization rates in the U.S., between July 2020 and April 2021. Lighter colors signify a smaller and darker colors are larger average hospitalization rate, ranging from 3.9% (Rhode Island) to 31.5% (Hawaii). The District of Columbia is an outlier with 78.4%, and not shown on the map

**FIGURE 2:**
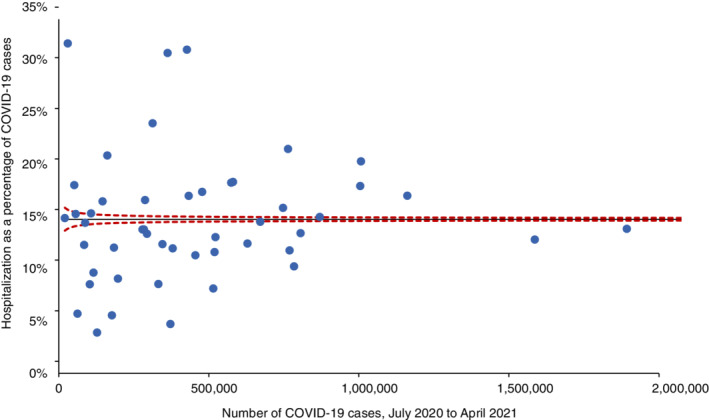
Funnel plot for COVID‐19 hospitalization rates at state level. In this funnel plot, every dot represents a U.S. state. Two states with more than 2 million cases (Texas, California) and the District of Columbia were excluded from the figure. Almost all states (except Vermont, Wyoming and Delaware) are outside of the 99% control limits (with Bonferroni correction; dashed line), that is, they are subject to special cause variation

The intraclass correlation coefficient in a one‐way random effects ANOVA (unconditional) model was ICC = .943, that is, along the timeline more than 94% of the variation in the hospitalization was between states, and only about 6% was within states; the variation between states was statistically significant (*r*_0_ = 108 240.019, *P* < .001). Likewise, the average COVID‐19 hospitalization rate, calculated as a percentage of total cases during July 2020 to April 2021 and shown in Figure 1, ranged from 3.9% (Rhode Island) to 31.5% (Hawaii). Most states were subject to special cause variation, as indicated by the funnel plot in Figure 2. Thus, it was deemed prudent to proceed with the longitudinal hierarchical model as specified above; Table 1 shows the model's fixed (Panel A) and random effects (Panel B). Hospitalization was higher in states with a higher percentage of Black (*β*_11_ = .002, *P* = .009) and AIAN persons (*β*_13_ = .003, *P* = .03). Conversely, lack of health insurance was related to lower hospitalization (*β*_16_ = −.005, *P* = .002), and so was a stronger social support system (*β*_113_ = −.016, *P* = .05). With respect to comorbidities, only diabetes showed a significant negative relationship to hospitalization (*β*_19_ = −.015, *P* = .04). States with higher government spending per capita also exhibited lower hospitalization (*β*_112_ = −.004, *P* = .02).

**TABLE 1:** Effects in the contextual model

Panel A: Fixed effects						
Fixed effect	*β*	*se*	95% CI	*df*	*P*‐value	Reliability
Mean country hospitalization, *π*_0_						
Intercept, *β*_00_	294.516	45.622	[205.097; 383.936]	50	< .001	1.000
For cases slope, *π*_1_						
Intercept, *β*_10_	.068	0.003	[0.061; 0.074]	37	< .001	0.623
Black, *β*_11_	.002	0.001	[0.001; 0.003]	37	.009	
Hispanic, *β*_12_	.001	0.001	[−0.001; 0.002]	37	.310	
AIAN, *β*_13_	.003	0.002	[0.000; 0.007]	37	.033	
Asian, *β*_14_	.000	0.002	[−0.004; 0.003]	37	.795	
Rural, *β*_15_	.000	0.000	[−0.001; 0.001]	37	.617	
Uninsured, *β*_16_	−.005	0.002	[−0.008; −0.002]	37	.002	
Median age, *β*_17_	−.004	0.002	[−0.008; 0.000]	37	.076	
Disabled, *β*_18_	.005	0.004	[−0.003; 0.012]	37	.239	
Diabetes, *β*_19_	−.015	0.007	[−0.028; −0.001]	37	.036	
Obese, *β*_110_	.000	0.002	[−0.003; 0.004]	37	.829	
Smoking, *β*_111_	.002	0.002	[−0.003; 0.006]	37	.470	
Govt. spending, *β*_112_	−.004	0.002	[−0.007; −0.001]	37	.024	
Social support, *β*_113_	−.016	0.008	[−0.031; 0.000]	37	.050	
For deaths slope, *π*_2_						
Intercept, *β*_20_	−.895	0.220	[−1.326; −0.464]	50	< .001	0.527

Panel B: Random effects					
Random effect	SD	Variance	*df*	*χ* ^2^	*P*‐value
Intercept, *r*_0_	329.013	108 250.038	25	49 274.465	< .001
Cases slope, *r*_1_	0.024	< 0.001	12	103.537	< .001
Deaths slope, *r*_2_	1.368	1.870	25	45.578	.007
Level 1, *e*	80.395	6463.427			

*Note*: This table shows the fixed and random effects in the longitudinal hierarchical model, estimated with restricted maximum likelihood and 7 iterations. The number of level‐1 and level‐2 records were 13 464 and 51, respectively. Robust standard errors (*se*) are reported.

The validity and robustness of the results were confirmed with a few tests. First, hospitals may have different admission thresholds depending on inpatient bed utilization and current or anticipated critical staffing shortages. Such information is available in HHS, but none of these variables entered the regression model with statistical significance. That is, hospital resource availability did not significantly influence COVID‐19 hospitalization. Second, deaths can be thought of as a final outcome of a small proportion of infections and hospitalizations, rather than as an indicator of the threat level. Removing the variable from the level‐1 equation confirmed the results with respect to direction and statistical significance; only diabetes and social support were no longer statistically significant. Third, removing the District of Columbia as an outlier (see Figure 1; now level‐1: 13 200, level‐2: 50 records) confirmed all result, with the exception of diabetes, *β*_19_ = −.012, *se* = 0.006, *df* = 36, *P* = .06. Fourth, multicollinearity can be a serious issue in epidemiological studies.^19^ Setting up all level‐1 regressions separately for all states resulted in a low average variance inflation factor of VIF = 1.991. At level 2, only the correlation between disabled and smoking was notable, *r* = .814, *P* < .001. More importantly, the automatic multicollinearity checks of HLM 7.3 did not identify any issues. Fifth, specification of a hierarchical model in HLM 7.3 with a longitudinal design where within‐state observations are thought of as actualization of a two‐level data structure requires similar covariance structures.^20^ The Durbin‐Watson statistics of the separate level‐1 regressions show that the temporal autocorrelations were not vastly different between states, mean *d* = 0.625, SD =  0.257.

## 3 DISCUSSION

States with a higher percentage of Black and AIAN persons had a disproportionally higher hospitalization for COVID‐19; these findings are consistent with previous studies showing disproportionate COVID‐19 incidence, hospitalization, and mortality among those racial/ethnic groups.^5^, ^6^, ^7^, ^8^ The health of these groups is affected by long‐standing, systemic inequities, including limited access to quality health care and education, over‐representation in essential jobs with less work‐from‐home flexibility, and possibilities for medical leave.^8^ Contrary to prior studies, the effect was not significant for Hispanic or Asian persons as well as for the median age.^7^, ^21^ The percentage of the rural population was not significant either.

The productivity of social capital, which comprises of all aspects of human relationships that produce benefits, is unequally distributed across the U.S.; it is important for understanding national challenges and creating effective policies.^12^ This study showed that states in which people enjoy more social support through family, friends, and neighbors are associated with lower demand for hospitalization; a withering associational life increases the need for external hospital care. People without health insurance coverage generally have worse access to health care than people who are insured.^10^ Correspondingly, COVID‐19 hospitalization was lower in states with a higher percentage of uninsured people. While good overall health is a general indicator for disease resistance, the health belief model suggests that a person's belief in a personal threat of a disease, together with faith in the effectiveness of behavioral recommendations, predicts the likelihood of the person adopting the recommendation.^1^ This was evidenced by states with higher levels of diabetes experiencing lower demand for hospitalization. Though, the prevalence of obesity, smoking, or disabilities did not show a statistically significant effect. Per capita direct spending by state and local governments varies widely across the U.S.; it both depends on state policy choices and external factors, such as geography, demographics, or history. In this study, higher per capita spending was negatively associated with hospitalization.

Effectively protecting the health of a country's population relies on having data to assess reasons for health disparities. Continued use of this study's model can help to identify health disparities experienced by certain racial/ethnic groups. Importantly, access to hospital care is also related to its affordability.

The findings in this report are subject to at least five limitations. First, given the ecological character of the study, conclusions at the individual level could not be drawn. Second, because the data source for COVID‐19 cases and hospitalizations does not allow linkage at person level, hospital readmissions could not be considered in this study. Third, the utilization of ambulatory care was not considered. Both persons with and without visit to an ambulatory care physician were included in the independent variable. The present analysis does not account for, inter alia, utilization of early sequenced multidrug therapy for COVID‐19. Because this approach is associated with 85% reductions in hospitalization, differences in access to this therapy may account for some of the differences observed.^22^, ^23^, ^24^ Fourth, the models did not control for other policies and measures, such as school closures, mobility restrictions, physical distancing recommendations, and vaccinations. It was assumed that these could not directly affect demand for hospitalization, but only indirectly via changes in the case count. Fifth, systemic inequities can disproportionally affect the social support structure. Being an ecological study, this could not be examined.

To summarize, this ecological investigation highlights that Black and AIAN persons sought hospitalization for COVID‐19 at higher rates than White persons. Conversely, lack of health insurance and a stronger social support system were related to a lower demand for hospitalization, reflecting differences in demand for medical care. With respect to comorbidities, only diabetes showed a negative relationship to hospitalization. States with higher government spending per capita exhibited lower demand for hospitalization. Such models are needed to forecast patient care demands, and allow for adequate staffing and resource allocation. In addition, in order to reach disproportionately affected groups and reduce the need for hospitalization for COVID‐19, it is important to prioritize health communication, prevention resources, policies (eg, work leave), and measures (eg, testing and vaccination). Such efforts are essential to address the drivers of disparities. Regional ecological data on COVID‐19 coupled with contextual information are important and effective sources for analyzing public health conditions. It allows to identify regional areas of inequity, communities affected the most, and build responses aimed at ensuring equitably accessible health prevention.

## CONFLICT OF INTEREST

The author is not aware of any competing interests.

## AUTHOR CONTRIBUTION

Conceptualization: Wolfgang Messner

Data curation: Wolfgang Messner

Formal analysis: Wolfgang Messner

Methodology: Wolfgang Messner

Writing‐original draft: Wolfgang Messner

## TRANSPARENCY STATEMENT

The author has read and approved the final version of the manuscript. Wolfgang Messner had full access to all of the data in this study and takes complete responsibility for the integrity of the data and the accuracy of the data analysis. He affirms that this manuscript is an honest, accurate, and transparent account of the study being reported; that no important aspects of the study have been omitted; and that any discrepancies from the study as planned (and, if relevant, registered) have been explained.

## ETHICS STATEMENT

No ethics approval was sought, because no humans or animals participated in this subject. Ethics approval or informed consent requirement were therefore not deemed to be applicable.

## Data Availability

The data that support the findings of this study are openly available in OSF at https://osf.io/wfj82/?view_only=504eb05dc3ae4639b36d867396a047ac. It is compiled and calculated from data publicly available in USAFacts at https://usafacts.org/visualizations/coronavirus-covid-19-spread-map, the U.S. Department of Health and Human Services (HHS) at https://healthdata.gov/Hospital/COVID-19-Reported-Patient-Impact-and-Hospital-Capa/g62h-syeh), and the Social Capital Project at https://www.jec.senate.gov/public/index.cfm/republicans/2018/4/the-geography-of-social-capital-in-america.
